# Method of Setting Environmental Administrative Fine Amounts

**DOI:** 10.3390/ijerph18095011

**Published:** 2021-05-09

**Authors:** Chang-Ying Hu, Shi-Hai Zhu

**Affiliations:** 1 School of Mathematics, Renmin University of China, Beijing 100872, China; huchangying@ruc.edu.cn; 2 Law School, Macao University of Science and Technology, Macao 999078, China

**Keywords:** environmental administrative fine, business operator’s interest, public interest, environmental protection

## Abstract

In China, there are currently different degrees of arbitrariness in setting environmental administrative fines, and in many areas the faults are not equal to the penalties. To construct a more reasonable and feasible environmental punishment strategy where violators are fined in accordance with the severity of their actions, we use mathematical models to determine the specific range of environmental administrative fines based on the idea of realizing the appropriate balance between the interest of the violators and those of the public, meanwhile, law enforcement officers are allowed to use their discretion within a certain range. We use an example to prove that the punishment scheme provided by our models can be used to more effectively supervise violators’ illegal behaviors than the penalty clause prescribed by law, and through sensitivity analysis and comparison, we prove that the described models are stable and feasible, and provide advantages over the existing methods. We hope our approach provides intellectual support for maintaining legal order, regulating the environmental administrative fine process, guiding business behaviors, and, most importantly, achieving the goal of protecting the environment.

## 1. Introduction

To achieve sustainable economic and social development, more atttention shall be paid to the ecological environment. At present, China’s seven major water systems (including the Liaohe River, Haihe River, Huaihe River, Yellow River, Songhua River, Pearl River, and Yangtze River) are polluted to varying degrees. Among them, more than 70% of the water in the first four rivers is polluted [1]. Most of the pollution in these rivers has been caused by the industrial discharge of substandard wastewater and solid refuse. Therefore, it is necessary to investigate and affix legal liability to those who cause environmental pollution and damage. The state has formulated environmental administrative penalties for those offending enterprises to prevent and reduce all kinds of polluting behaviors. With the revision of China’s Environmental Protection Law of 2014, the authority enforcing the ecological and environmental administrations in various provinces and cities was greatly expanded, and the amount of fine and punishment measures were increased significantly. In 2018, the average environmental administrative fine in China was RMB 15.28 billion Yuan, an increase of 32% over the previous year [2]. After investigating a large number of environmental cases, we found different degrees of arbitrariness in setting fine amount existed and caused inequality between faults and punishment in many provinces, despite the large collective amount of fine. Law enforcement officials sometimes relied on subjective judgment when assigning penalty amounts; sometimes, the amount of the penalty was set with reference to similar cases, without specific or scientific principles supporting the punishment. Hence, penalties sometimes were either too light or too heavy, meaning the fine was often inappropriate.

To be specific, inappropriate punishments manifest in two aspects: insufficient punishment and excessive punishment. Examples of insufficient punishment can be found in many provinces in China, where even the highest environmental pollution fine is far less than that imposed in foreign countries on similar polluters. In some cities, some companies that have caused very serious pollution were fined less than 200,000 yuan, or even less than 10,000 yuan in some cases. This may have a deterrent effect on small- and medium-sized enterprises, but for large enterprises, the fines have little influence, so they do not show respect for the authority and dignity of the law [3]. Regarding the practice of excessive punishment, believing that an administrative punishment cannot stop the behavior of the polluters, there is a strong voice in society to punish the violators into bankruptcy. In practice, there are exorbitantly high fines in a few areas [4]. Increasing the fine may deter the offenders and reduce the proportion of violators who break the law; however, an excessive fine will also cause some problems, such as working against the protection of property rights, endangering the personal independence of business operators, and ignoring the social costs of a bankruptcy [5]. Excessive fines may also result in serious business failure, withdrawal, or economic recession, as a result, hindering economic development and weakening the power of the state. Hence, both insufficient and excessive fines have negative effects. Therefore, an appropriate penalty strategy is the key principle that the administrative subject should follow when setting fine amounts. An appropriate fine not only guarantees the appropriate punishment of violators, thus effectively preventing them from breaking the law, but also ensures that the penalty amount is within the scope of the violators’ bearing, which avoids the negative impact(s) on economic development to a large extent.

Then, the question arises as to how to set an appropriate fine amount? First, we know that inappropriate punishment stems from the discretion of law enforcement departments and the lack of a set of scientific and specific punishment methods. Thus, to construct a proper method of punishment, the discretion of law enforcement departments should first be appropriately regulated. On 22 May 2019, the Ministry of Ecology and Environment of China issued “The Guiding Opinions on Further Standardizing the Application of Discretion of Environmental Administrative Penalty *“*(hereafter referred to as the Opinions) [6]. China wants to regulate the discretion of environmental protection agencies and to establish a scientific and feasible method to determine the amount of an administrative fine. However, the Opinions did not outline a specific implementation plan, and there are still some problems that need to be solved, such as the scope of effectiveness, technical issues, and how to deal with conflicts between the superior and subordinate departments of the local governments [7]. Therefore, how to scientifically use this discretion to construct a reasonable and feasible punishment method shall be further explored.

This problem has aroused academic interest, and many scholars have conducted related research in these years, intending to help the government establish scientific punishment methods. However, until now, most studies only focused on the analysis of the factors influencing punishment and the ability to pay. For example, Neda Oljaca et al. used regression analysis to study 140 water pollution punishment cases in Georgia from 1986 to 1995, and found that the amount of administrative fine was positively related to the attitude of operators, the number of violations, and the degree of pollution [8]. Dufau thought that it was necessary to consider the ability to pay when setting the penalty amount, and that it was difficult to assign a fine exceeding the payment capacity. Therefore, Dufau described the upper limit of the penalty amount using a formula [9]. As more and more people got involved in research on this problem, some specific punishment methods were provided, such as using Han De formula to set administrative fines [10], the multiple-rate fine method [11], the standard administrative penalty formula for business-oriented violations [12,13], grid techniques [14,15] and so on.

These methods have their merits. For example, by analyzing the factors that influence punishment, law enforcement officials know what issues should be paid attention to in the process of enforcing the punishment. Some research methods provided by the existing research laid the foundations for the government to assign punishment. However, there are also limitations: The first one is that some models do not fit the actual problem. e.g., using the Markov model to study the determination of an administrative fine is more suitable for cases of random illegal acts, but not for some cases of the intentional violation of the law for the sake of greater interest. To reduce costs and increase profits, many enterprises in China deliberately violate environmental laws in the hope that they will not be discovered. In this case, it is not appropriate to use a random model. The second limitation is that some factors are hard to measure. For example, some variables in the Han De formula are qualitative variables that not easy to be quantified, which poses challenges with using the Han De formula in practice. The third one is that the key factor is difficult to determine. The multiple-rate penalty method is applied to environmental administrative fines, but it is difficult to determine the appropriate multiplier, as there are considerable differences between various fields. In some areas, the amount of the fine is 15%–20% of the base amount, yet in other areas, it is 1–5 times of the base data or even 5–10 times higher. The huge difference of multiples not only exists between different fields, but also in the same field at times [11]. With grid technology, due to its own defects, it cannot be used to fully evaluate all the relevant factors. The combination is singular, but the restriction on discretion is still too broad, so it cannot achieve the expected goal of the discretionary benchmark [16].

In short, the academic community has had large amounts of beneficial discussion on the method of setting the amount of administrative fines, but some deficiencies have not been overcome. What’s more, most authors only considered the impact of enterprises’ polluting behaviors on the environment and the public, and failed to take into account the interest and development of enterprises at the same time. In fact, when studying environmental punishment, we should not unilaterally consider how to punish enterprises to protect the environment and the public interest, but also take into account the development of the enterprises and the economic development of the whole country. Thus, given this, we aimed to construct mathematical methods for determining the appropriate range of the amounts of environmental administrative fines considering the interest of both the public and the enterprises or individuals that violate the rules. At the same time, we expect the government to exercise discretion within a certain scope to ensure the punishment scheme is reasonable and feasible.

## 2. Mathematical Models for Determining the Amount Range of Environmental Administrative Fines

When it comes to the punishment for environmental harm, the general principle is that the person who destroys is responsible, and punishments should be equal to the faults. Therefore, the punishments should differ according to the degrees of pollution damage. Thus, the question arises as to how to determine the amount of fine according to the degrees of damage. As a basic methodology of legal interpretation, interest measurement has been widely used in the field of administrative law, and constitutes the core connotation of administrative discretion [16]. Take businesses as an example, at the micro level, the best method to determine the amount of fines for an enterprise that violates the rules should take into account the interests of both the enterprise and the public. The profit of an enterprise is often related to how it operates. Sometimes, an enterprise may damage the environment to obtain extra benefits, such as overusing natural resources, or discharging industrial wastewater into nearby rivers without sewage treatment to reduce costs. In this situation, the more resources were used, the more benefits were gained, and the more substandard wastewater was discharged, the more cost savings were achieved. Hence, the more seriously they destroy the environment, the greater the benefits they presumably obtain, so we assume that the benefit (or interest) function of the enterprise increases with the increase in the degree of illegality. In a given period (a week or a month), if the enterprise does not damage the environment, i.e., using resources rationally and discharging waste water, gas, and residue according to environmental standards, the benefit value is a (a>0). Although the enterprise owner or the business operator knows that violation of the environmental law may be punished, as long as the expected punishment is less than the illegal income, the enterprise owner may choose to violate the law [16]. We assumed that there are multiple levels of illegal actions, where level 1 is a lower-level violation, level 2 is a higher-level violation than level 1, and so on. Let x (x > 0 continuous variable) be the violation level or illegality degree of the enterprise, and different value ranges of *x* correspond to different levels of illegality. Let b be the average cost of solving or eliminating one level of violation, i.e., if the illegal degree increases from level 1 to level 3, the enterprise adds two levels of violations and may save cost 2b. Then if the enterprise chooses to violate the environmental law and its violation level is x, it will reduce the pollution treatment cost bx and its benefit function will be a+bx (where a and b can be obtained from the production data of the enterprise, and they are all constants). However, if there is an illegal action, the government may find and punish it. Let us assume that the probability of a firm’s wrongdoing being discovered is p (0<p≤1) (here we assume that the illegal action must be found, so p≠0). Let y (y≥ 0,continuous variable) be the fine amount, then the expected average income (or interest) function of the enterprise can be written as:

(1) f(x,y)=p(a+bx−y)+(1−p)(a+bx) (x>0,y>0)

where in Equation (1), we take the expected value of the revenue function as the objective function, which is a common definition mode in economics.

Polluting (destroying) the environment inevitably infringes on the public interest, because when illegal activity occurs, it will negatively impact society and damage the public’s interest. The higher the degree of the violation, the greater the harm to the public. For example, some companies dump untreated industrial waste water into rivers, which pollutes drinking water sources, and brings some diseases to people. If the water is used to irrigate fields and grasslands, it will pollute food and livestock, causing great harm to public health; the more pollution companies cause, the more damage the public suffers. In China, treating and repairing environmental damage is often the duty of the government, and the government has to use public finance (mainly funded by fines for violations) to fulfill this obligation. Hence, China’s environmental penalties also play an important role in fixing environmental damage [17,18]. The larger the fine, the more compensation the public receives. Hence, the public interest function will decrease when the illegality degree x increases, and will increase when the fine amount y increases, so the public interest function can be set as:

(2) $$F\left(x,y\right)=k\frac{y}{x},$$

where 0<k≤1 is the proportion of the penalty that reimburses the public for its loss. Here, we assume that the function F(x,y) is a nonlinear function of x rather than a linear function because it is generally impossible for the level of violation (or illegality degree) x to be zero, especially in the case of industrial wastewater, which generally could not be treated into pure water. The inverse function $\frac{1}{x}\;$can be more suitable to simulate the relationship between the public interest and the illegality degree x. Next, we will discuss how these two functions in Equations (1) and (2) change with respect to these two variables x and y.

**Figure 1:**
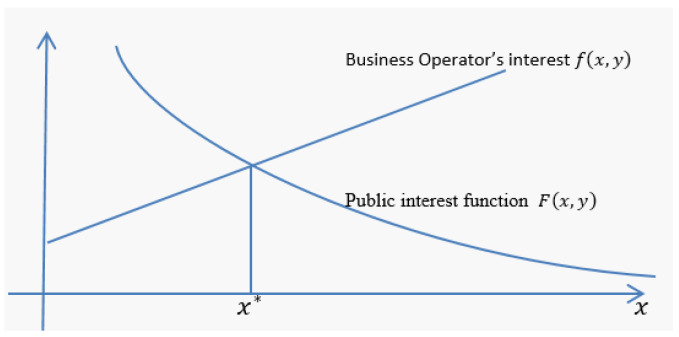
Public interest function and business operator’s interest function under given penalty y.

(a) First, let us observe how f(x,y) and F(x,y) change when we fix the fine amount y. Given fine y, the public interest function $F\left(x,y\right)=k\frac{y}{x}\;$ is a decreasing function of the illegality degree x, while the business operator’s interest function f(x,y)=p(a+bx−y)+(1−p)(a+bx) is an increasing function of x (see Figure 1).

In Figure 1, the intersection point $x^{*}$ is called the balance point of the public interest function and the business operator’s interest function; At this point, the interests of both are the same. The public interest is greater than that of the business operator when the illegality degree x is less than the balance point $x^{*}\;(x<x^{*})$; In this case, the public interest can be safeguarded. However, when the illegality degree x is larger than $x^{*}\;(x>x^{*}),\;$the public interest will decline rapidly and will be lower than the operator’s interest, which means the operator maybe damage public interest. As a result, the public’s interest are not always guaranteed. Therefore, if the government fixes the penalty amount y, only when the operator’s illegality degree x is less than the balance point $x^{*}$ can we defend the public’s interest. Once the illegality degree x breaks the balance, the government must adjust the amount of punishment in a timely manner according to the level of the violation; Otherwise, the public’s interest will be hurt. Then, how should the government adjust the penalty according to the violation level of the enterprise?

**Figure 2:**
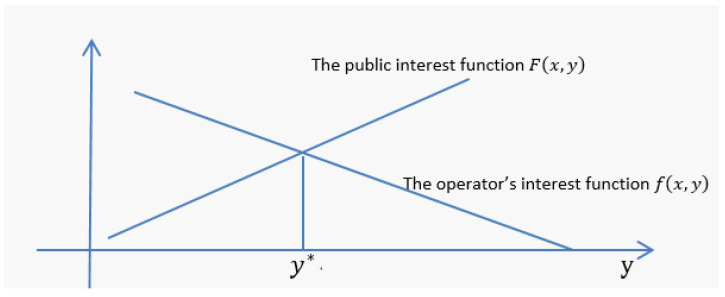
The public interest function and the operator’s interest function under the given illegal degree x.

(b) Now, let us observe how these two functions change by fixing the degree of violation x. Given x, F(x,y) is an increasing function of the fine amount y, while f(x,y) is a decreasing function of y, as shown in Figure 2.

The intersection point $y^{*}$ of the two functions is also an equilibrium point, where the interests of the public and the enterprise are same for a given degree of violation. When $y<y^{*}$, i.e., the fine amount y is less than equilibrium point $\;y^{*}$, the enterprise’s interest will be higher than the public interest; In this case, the smaller the fine, the greater the loss of public interest. Only when $y\;\geq y^{*}$ will the public interest be protected. Therefore, if the public interest is not lower than that of the enterprise, $y^{*}$ is the minimum penalty point. Next, we will calculate $y^{*}\;$and observe the relationship between $y^{*}$ and the degree of violation x (see Equations (3)–(5)).

Now, let us calculate the equilibrium point $y^{*}$. Let f (x, y)=F (x, y), i.e.,

(3) $$p\left(a+bx-y\right)+\left(1-p\right)\left(a+bx\right)=k\frac{y}{x}$$

We obtain:

(4) $$y^{*}=\frac{\left(a+bx\right)x}{k+px}\;\left(k+px\neq 0\right)$$

$y^{*}\;$is a function of the illegality degree x, and

(5) $$\frac{{\mathrm{dy}}^{*}}{dx}={\left(\frac{\left(a+bx\right)x}{k+px}\right)}^{\prime }=\frac{k\left(a+2bx\right)+pbx^{2}}{{\left(k+px\right)}^{2}}.$$

Since k,a,b,x, and p are all nonnegative, we get $\frac{{\mathrm{dy}}^{*}}{dx}\geq 0$, which means $y^{*}$ is an increasing function of x, and the higher the illegality degree *x*, the higher the fine $y^{*}$.

Now, let’s consider the maximum penalty point. Since the maximum punishment had better not be bankruptcy, then let the interest of the enterprise in this period is zero, thereby obtaining the maximum penalty point (see (6) and (7)). Let the interest function f(x,y)=0, i.e.,

(6) f (x, y)=p(a+bx−y)+(1−p)(a+bx)=a+bx−py=0.

We obtain:

(7) $$\;\hat{y}=\frac{a+bx\;}{p}\;\left(p\neq 0\right)$$

which means the enterprise’s benefit is zero when we set the amount of the fine $y=\;\hat{y}$. Compare $\;\hat{y}$ and $y^{*}$, and by substituting $\;\hat{y}\;$into $y^{*}$, we get Equation (8):

(8) $$y^{*}=\frac{\left(a+bx\right)x}{k+px}=\frac{px}{k+px}\;\frac{a+bx}{p}=\frac{px}{k+px}\;\hat{y}\;.$$

Because $k>0,\;0<p\leq 1,so\;\frac{px}{k+px}\leq 1$. As a result,

$$y^{*}\leq \hat{y}$$;

that is, the minimum fine amount $y^{*}$ will not exceed $\;\hat{y},\;$which makes the profit of the enterprise zero. Hence, we obtain a corresponding fine range: $\left[y^{*},\;\hat{y}\right],\;\mathrm{where}\;y^{*}$ is the lower limit of the fine and $\hat{y}$ is the upper limit of the fine for the given illegality degree x. In this way, for each illegality degree x, there is a corresponding penalty range $\left[y^{*},\;\hat{y}\right]$; within this range, the government can use its discretion to determine the exact amount of penalty for this level of violation. Here, we take$\;\hat{y}$, the point at which the profit of the operator is zero, as the upper limit, rather than the amount that would force the offender to close down, because the operator also needs to survive and develop and the national economy needs to move forward. We should not achieve economic development at the expense of harming the environment; similarly, we cannot only focus on environmental protection while ignoring economic development. Since there is a maximum penalty $\;\hat{y}\;$for each illegality degree x, when the illegality degree x reaches the maximum (denoted as $x_{max}$), the corresponding upper limit of punishment (recorded as ${\hat{y}}_{max}$) is the highest penalty value of all. Here, ${\hat{y}}_{max}$ would impose a serious restriction on the business and would discourage it from further violations.

Now, let us answer the question in (a): How does the government determine the amount of punishment based on the violation levels of the enterprise? The government only needs to determine the illegality degree x of the offending company, which can be determined by measuring emissions. Then using $\left[y^{*},\;\hat{y}\right]\;$ to get the minimum penalty $y^{*}$ and the maximum penalty$\;\hat{y}\;$corresponding to this level x. The specific amount can be determined by the law enforcement personnel to exercise discretion. They can use the grid technology, or refer to multiple-rate fine method, or just make a decision based on the number of times the enterprise violates the rules, the consequences caused, and the attitude of the enterprise. In the next part, we will use an example to show how to determine every penalty range $\left[y^{*},\;\hat{y}\right]$ based on each level of violation.

In this way, we have obtained a new penalty strategy by studying the relationship between the violator’s interest and the public interest. For this strategy, only the level of violation x of the enterprise needs to be determined, and then the specific punishment range $\left[y^{*},\;\hat{y}\right]\;$can be determined. This method has the following advantages: First, this method can determine a specific range of penalty amounts according to the degree of violation for an administrative subject to impose administrative fines on offenders. This not only protects the interest of the public, but also takes into account the interest of the operators. Second, our method takes into account the enterprise’s ability to pay (the maximum penalty is to make the company’s profit for this period zero), and in the meantime, prompts business operators to pay more attention to the relationship between their profit and their illegal activities, so it may be able to prevent illegal activities in advance to a large extent. Third, this method mainly takes the degree of violation x as the factor to determine the range of environmental administrative fine amounts, and different degree of violation corresponds to different fine range. This system design not only further implements the principle of punishments equaling faults, but also permits the administrative subject to use its discretionary power within a certain range, simultaneously limiting the arbitrariness of fines caused by the excessive discretion of the administrative subject. Hence, this method can be used to effectively avoid many disadvantages, such as eliminating the diversity of administrative affairs, ignoring subjective initiative of law officers, reducing the administrative efficiency and hindering the realization of administrative case justice [19]. We will further demonstrate the advantages of this approach in the following case study.

## 3. Case Study

From February to April 2017, the Ministry of Environmental Protection of the State carried out a special inspection on groundwater pollution caused by the sewage from enterprises in the North China Plain. A total of 25,875 enterprises were investigated and 558 environmental violations were found: 54 enterprises were found to have discharged, transported, or stored sewage using seepage wells, seepage pits, or ditches and ponds without antileakage measures. More than 423 enterprises were ordered to rectify the situation within a prescribed time, fines were imposed upon 88 enterprises, 80 illegal enterprises were put on file, and more than 6180 enterprises were ordered to carry out appropriate sewage treatment procedures (data from Chinanews Network [20]). At present, the main bases for fines are articles 73 and 74 of the Water Pollution Prevention and Control Law revised in 2008 [21]. However, the fine amounts are too low to produce a practical effect. This shows that the punishment standard of the Water Pollution Prevention and Control Law has not kept pace with the times. Is it necessary to amend the Water Pollution Prevention and Control Law in order to allow for the imposition of heavy fines for all enterprises to prevent environmental pollution? It may not be wise or ideal because we must consider the business operator’s ability to pay the fine. The fine will have no practical significance if the amount of the fine is beyond the enterprise’s ability to pay. Hence, the amount of the fine should be consistent with the level of the violation, should not exceed the tolerable range of enterprises.

Let us consider a leather factory in the north of China that was fined in 2017 as an example (the name of the factory is hidden here) to illustrate how to formulate the punishment strategy, so that the factory can actively control pollution and discharge wastewater according to the statutory standards.

**Figure 3:**
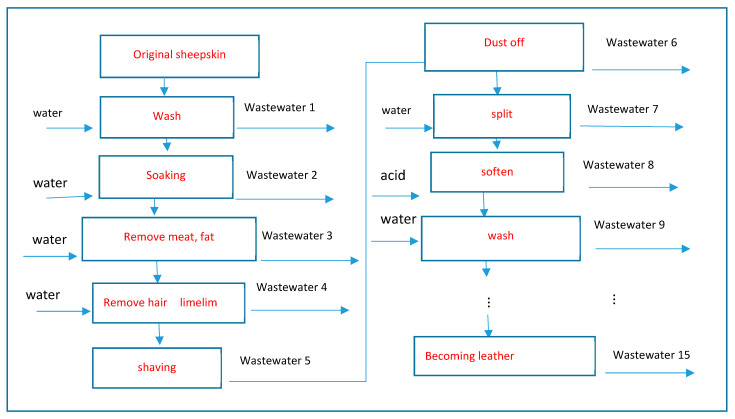
Leather Production Process.

The leather factory was mainly engaged in the leather processing and manufacturing business. It takes 25 processes for a piece of ordinary sheepskin to be processed into finished leather (see Figure 3).

Through those processes, 15 kinds of wastes are produced and discharged along with the wastewater. Therefore, the wastewater contains dozens of harmful substances such as formaldehyde, acetone, chloride, preservatives, limestone, etc. These substances make the groundwater contain a large number of anions, such as $\mathrm{HC}O_{3}^{-}$, $SO_{4}^{2-}$, $NO_{3}^{-},\;\;\mathrm{and}\;Cl^{-}$, and cations, such as $C_{a}^{2+},\;\;N_{a}^{+},\;and\;M_{g}^{2+}$, which make the groundwater acidic or alkaline [22] and harmful to human health, crop production, animal husbandry, and so on [23]. Hence, the wastewater should be purified before it is discharged into the outside world. Generally, this kind of wastewater must undergo at least eight procedures before it can attain the standard for safe discharge, and each procedure requires special locations, equipments, chemical reagents, and professional and technical personnel. These requirements mean that the cost for cleaning the wastewater is high. Unless the government supervises the production process, the factory may not clean the wastewater in order to avoid the expense. Therefore, the government must compel the factory to purify the sewage by irregular inspections.

Through the factory research, we know that the leather factory produces about 6 tons of wastewater every week, and the wastewater must be discharged to the outside world in a timely fashion; otherwise, there will be no place for the next week’s wastewater. This kind of sewage needs eight purification procedures before it is discharged, and each procedure corresponds to a wastewater level, so eight purification procedures means eight wastewater levels, denoted by x, 0<x≤8, where 0←x≤0.1 indicates that the wastewater treatment is qualified, i.e., all eight procedures have been completed, and the treated wastewater reaches the national discharge standard; 0.1<x≤1 indicates that the waste water level is level 1, i.e., there is a procedure that was not carried out or was not carried out thoroughly, and the wastewater does not meet the discharge standard; i−1<x≤i, i=2,3,4,⋯8  indicates that the wastewater level is level i, which means that there are i processes that were not carried out or the processes were carried out, but the amounts of some pollutants were still higher than the discharge limits; and x=8 indicates that none of the eight processes were carried out.

According to the factory’s sales data, the profit for one week after strictly cleaning the wastewater is about a=50,000 yuan (here, a is an estimated integer according to the manufacturer’s data for the convenience of calculation), and the average cost of each purification procedure is b=2000 yuan. Generally, the cost of treating different pollutants is different, but for convenience, we use the average cost of the eight processes (*b* = 2000 is an estimated value given by the manufacturer). That is, if one purification procedure is removed, 2000 yuan will be saved. Assume that the Environmental Protection Department will perform irregular inspections on the factory, and the probability of finding polluting behavior is p, where p can be determined according to the ratio of the number of illegal emissions to the total number of spot checks in the past. Usually, we set p=0.5 (the median). If the factory is found to have illegally discharged substandard sewage, it will be fined according to the grades of sewage. Let y be the amount of the fine, then the average profit function of the factory for each week is:

(9) $$\begin{array}{ll} f\left(x,y\right) & =p\left(a+bx-y\right)+\left(1-p\right)\left(a+bx\right)\\ & =p\left(50000+2000x-y\right)+\left(1-p\right)\left(50000+2000x\right)\\ & =50000+2000x-0.5y \end{array}$$

From the perspective of the public, the illegal discharge of substandard wastewater by the factory will harm the public’s interest, such as damaging people’s health and polluting food and drinking water. Thus, the government usually decides to use part of the fine to compensate the local public for their loss and to repair the function of the polluted land. Setting  k=0.9, as the local government reported spending more than 90% of fines on repairing environmental damage, then the public interest function is

(10) $$F\left(x,y\right)=k\frac{y}{x}=\frac{0.9y}{x}.$$

According to Equations (4), (7), (9) and (10) we can calculate the minimum penalty amount $y^{*}=\frac{\left(a+bx\right)x}{k+px}=\frac{\left(50000+2000x\right)x}{0.9+0.5x}$, and the maximum penalty amount $\hat{y}=\frac{50000+2000x}{0.5}$.

For example, when the wastewater level is level 1, we substitute x=1  into $y^{*}\;$and $\hat{y}$: $y^{*}=\frac{\left(50000+2000\times 1\right)\times 1}{0.9+0.5\times 1}=37142.857$; $\hat{y}=\frac{50000+2000\times 1}{0.5}=104000$, therefore, the penalty range of level 1 is [37142.857, 104000]. Similarly, we can get the punishment ranges corresponding to other wastewater levels, as shown in the Table 1 below.

From the above table, we drew the following conclusions: First, for given *p* and k, the penalty amount y only varies with x, meaning that the level of wastewater x is the main influencing factor. Generally, it is easy to determine the discharge level of wastewater, meaning that the model given above is easy to apply. Second, for each wastewater level x, a penalty amount range $\left[y^{*},\;\hat{y}\right]$ is provided, and in this interval, the environmental authorities can use discretion to assign more reasonable punishment strategy based on the performance of enterprises. For example, assuming that the illegal degree of the factory is 3, that is, the factory had three procedures for the treatment of the wastewater that did not meet the national discharge standard, the law enforcement officer can set the fine amount in [70,000, 112,000]. The specific penalty amount is determined according to the content of the corresponding pollutants in the wastewater, the number of violations, and the attitude of the violators and so on. The maximum fine amount can be $\hat{y}$ = 112,000 yuan. Third, if fixing the penalty amount $y^{*},$ $f\left(x,y^{*}\right)$ and $F\left(x,y^{*}\right)$ both decrease as x increases, that is, a high degree of illegal activities is not only unfavorable to the public, but also to the factory. Hence, the factory is reluctant to offend the public by increasing their level of violation.

## 4. Sensitivity Analysis and Comparison

### 4.1. Sensitivity Analysis

We use mathematical models to calculate different penalty ranges according to different pollution levels of the enterprise. Now let’s look at the reliability of the models. In other words, parameters a, b, p,and k are used in the models. These parameters are generally obtained from the manufacturer’s own report and the government reports, but if these parameters are inaccurate, we want to know how much the penalty amount would be affected. Here, k is a relatively stable number, which can be verified by government reports; therefore, we discuss the influence of other parameters on the penalty amount $y^{*}$ and $\hat{y}$.

From Equations (4) and (7), we know that$\;y^{*}=\frac{\left(a+bx\right)x}{k+px}\;\left(k+px\neq 0\right)\;\mathrm{and}\;\hat{y}=\frac{a+bx}{p}$. We fix the illegality degree x and pollution control cost b, then calculate the elasticity of $y^{*}\;and\;\hat{y}$ with respect to a:

(11) $$E_{y^{*}}\left(a\right)=\frac{dy^{*}}{da}\;\frac{a}{y^{*}}=\frac{x}{k+px}\times \frac{a}{\frac{\left(a+bx\right)x}{k+px}}=\frac{a}{a+bx}\;;$$

(12) $$E_{\hat{y}}\left(a\right)=\frac{d\hat{y}}{da}\;\frac{a}{\hat{y}}=\frac{1}{p}\;\frac{a}{\frac{a+bx}{p}}=\frac{a}{a+bx}$$

We calculate the elasticity for each pollution level. For example, we set x=1 and b=2000, then at a= 50,000, according to Equations (11) and (12) we have $E_{y^{*}}=E_{\hat{y}}=\frac{50000}{50000+2000}\approx 0.96$, which means that the profit value a of the enterprise increases or decreases 1% from 50,000, so $y^{*}$ and $\hat{y}\;$will increase or decreases about 0.96%, which are less than 1%. This shows that $y^{*}\;and\;\hat{y}\;$are not much affected by a. For x=2,3,⋯8, parameter a has even less of an effect on $y^{*}$ and $\hat{y}$.

Next, we consider parameter b, which is the most likely parameter to be inaccurate because the cost b is related to the enterprise’s equipment, the size of the site, the ability of technical personnel, and the attitude of the enterprise:

(13) $$E_{y^{*}}\left(b\right)=\frac{dy^{*}}{db}\;\frac{b}{y^{*}}=\frac{x^{2}}{k+px}\times \frac{b}{\frac{\left(a+bx\right)x}{k+px}}=\frac{bx}{a+bx};$$

(14) $$E_{\hat{y}}\left(b\right)=\frac{d\hat{y}}{db}\;\frac{b}{\hat{y}}=\frac{1}{p}\;\frac{b}{\frac{a+bx}{p}}=\frac{bx}{a+bx}$$

We set x=1 and a=50000, then at b=2000, according to (13) and (14) we have $E_{y^{*}}\left(b\right)=E_{\hat{y}}\left(b\right)\approx 0.038\%$. This shows that $y^{*}\;and\;\hat{y}\;$ are almost unaffected by b. For other illegal degrees x, the same conclusion will be obtained.

For the probability p, we consider the elasticity:

(15) $$E_{y^{*}}\left(p\right)=\frac{dy^{*}}{dp}\;\frac{p}{y^{*}}=-\frac{\left(a+bx\right)x^{2}}{{\left(k+px\right)}^{2}}\times \frac{p}{\frac{\left(a+bx\right)x}{k+px}}=-\frac{px}{k+px};$$

(16) $$E_{\hat{y}}\left(p\right)=\frac{d\hat{y}}{dp}\;\frac{p}{\hat{y}}=-\frac{a+bx}{p^{2}}\;\frac{p}{\frac{a+bx}{p}}=-1\;$$

In Equations (15) and (16), we set x=1 and k=0.9, then at p=0.5, $E_{y^{*}}\left(p\right)=-0.35,\;E_{\hat{y}}\left(p\right)=-1$, which means that the probability of environmentally harmful actions being found increases every 1% from 0.5, $y^{*}$ decreases 0.35% and$\;\hat{y}$ decreases 1%; for every 1% decrease from 0.5, $y^{*}$ and$\;\hat{y\;}$increase by 0.35% and 1% respectively. Hence, $y^{*}$ and$\;\hat{y\;}$ are very little affected by p.

To summarize, both $y^{*}$ and$\;\hat{y\;}$ given by the models are stable and feasible because they are little influenced by the parameters. Even if these parameters are not very accurate, the solution given by the models still provides reference values.

### 4.2. Comparison with Existing Punishment Methods

Our study is different from the existing studies on environmental-related fines. We mainly adopt investigation methods, mathematical model analysis method and cross research method to study environmental punishment. But the existing literature mainly used qualitative analysis method and empirical analysis method. We mainly carry out theoretical research, and the existing literature is more empirical research. We assign fines based on the levels of violation, which are quantitative data and can be obtained from the test reports of the environmental protection department. The penalties previously given out were related to some qualitative factors that were difficult to measure, such as the attitude and honesty of the violator. Some methods such as the Han De formula, grid technology, and multiplier-rate method also depend on many qualitative factors, so there is no method of comparing the fine amount for the same level of pollution. Therefore, we only compare our results with the penalty policy stipulated by law. According to article 74 of the Water Pollution Prevention and Control Law enacted in 2008 (WPPCL of 2008), if a factory’s wastewater level is level 1, a fine of more than twice and less than five times the cost of pollutant discharge fee shall be imposed. Now, the pollutant discharge fee for level 1 is 2000 yuan, then the penalty amount shall be 4000 to 10,000 yuan according to the WPPCL of 2008, but the penalty range given by our model is 37,142 yuan to 104,000 yuan, far higher than the legal fine. In the groundwater pollution inspection of the North China Plain in 2017, the pollution level of most punished enterprises was level 4–8. Taking level 6 as an example, and the punishment range stipulated by law is [24,000, 60,000], but the punishment range given by our model is [101,818, 128,000]. The punishment imposed by the WPPCL of 2008 is too light, which may lead to repeated illegal acts. For this tannery, according to our punishment strategy, eliminating one level of pollution treatment can save 2000 yuan, but once discovered, the minimum fine is 37,412 yuan, so the factory will dare not choose to risk violations. Therefore, the method described in this paper will more effectively compel violators to take the initiative to implement pollution control measures.

## 5. Significance and Shortcomings of This Article

China has a long history of environmental problems. Since the founding of the People’s Republic of China, in order to rapidly improve its national strength, China has developed its economy at the cost of the environment, but with increasing environmental pollution, especially pollution harmed food production and people’s health became increasingly obvious, people began to pay attention to environmental problems, and the government began to issue environmental regulations to limit the pollution caused by enterprises or individuals and punish polluters. From the historical evolution of environmental administrative punishment legislation in China, there have been many and frequent law revisions, but environmental problems are still serious because the environmental legal responsibility system overemphasizes the rigid force of the administrative punishment, but neglects the flexibility within the administrative penalty system [24]. Because the violation of environmental regulations is determined by many uncertain and unquantifiable factors, it is hard to establish uniform, scientific, and feasible penalty standards. Hence, environmental law can only describe a general scope of punishment, and the final amount of a fine is decided by law enforcement personnel according to the situation. As a result, the amount of the penalty is often arbitrary, and China’s environmental administrative punishment amounts are erratic. The proposed method of determining the amount of environmental administrative penalty in this article emphasizes the authority of environmental law while considering the internal flexible function of the administrative penalty system. It can help law enforcers to assign different punishments according to different degrees of violation, while giving law enforcers certain discretion to make the punishment more reasonable and feasible. In addition, from the above example, the business operator will not readily break the law if the government supervises the operator according to our penalty strategy. Even if the operator violates the law, they dare not to increase the illegality degree lightly; otherwise, they will face the risk of zero profit or even business suspension. Therefore, this study is of great significance for China to help guide further clarifications of environmental penalty standards, and to formulate clear environmental laws and regulations. Moreover, the legislation based on this method of determining the amount of fines provides guidance for the behavior of operators, and forces the companies to focus on the costs of breaking the law while pursuing profits, so it provides a forward-looking function in protecting the environment.

This method also has its drawbacks. As it was difficult to obtain data regarding the amount of the punishment, the pollution level, and public interest, it was impossible to simulate the enterprise interest function and the public interest function with data, which seems the models in this article lack of data support. However, it is valuable for us to define these two models with the method commonly used in economics. Another drawback is that the study is not comprehensive enough. We presented our penalty strategy to the owner of this leather factory. He was reluctant to accept the fine strategy and said he was frustrated that the government is always ready to punish them without providing any help. Therefore, we cannot punish blindly, and should give rewards to enterprises who meet the environmental standards. Only when rewards and punishments are applied together can we achieve the goal of protecting the environment without harming the enthusiasm of enterprises. Therefore, our next research target is to establish mathematical models with which to study environmental policy involving both rewards and punishments. We hope to guide enterprises to actively control environmental pollution, and finally achieve the goal of stable production and environmental protection.

## 6. Conclusions

Scientific determination of the amount of an environmental administrative fine is necessary and meaningful to realize the rule of administrative law. In this paper, through the investigation of previous cases of environmental administrative punishment, we adopt quantitative analysis method and cross research method to study environmental punishment. Based on China’s existing practical problems including the setting of penalty benchmarks being too arbitrary, the punishment not producing effective deterrence, or exorbitantly high fines sending companies to extinction, and so on, we discussed a method of determining the amounts of environmental administrative penalties by using mathematical models. The method provides a specific penalty range according to the degree of illegality based on the concept of a moderate balance between the interests of the business operator and those of the public. This method is easy to operate and apply. It ensures not only necessary punishment for violators, but also that the amount of punishment is within the scope of the violator’s tolerance, thus minimizing the negative impact of the environmental administrative fine on economic development. Notably, this method limits the arbitrariness of discretionary power of the administrative subject, and at the same time, comprehensively considers the discretionary power of the law enforcers in various complex situations. Law enforcement officers are allowed to have discretion within certain limits, which is necessary for the actual enforcement process. In short, the method described above can be used by the administrative subject to determine a more reasonable penalty amount within a defined range, to provide guidance for the operator on how to operate legally, and, to a large extent, to prevent illegal acts in advance, ultimately protecting our environment. However, the research also has some shortcomings. Firstly, the interest function models lack data support; Secondly, incentive measures are not considered; the given strategy may discourage enterprises’ enthusiasm for production. We hope to overcome these shortcomings in the follow-up research.

Our model has a wide range of applicability, not only for environmental administrative fines, but also for dealing with other business administrative violations. This method can be applied to any situation involving enterprises or individuals who seek to improve their interests by damaging others’ interests. The practical operation of this method is strong since general software, such as MATLAB and Excel, as well as common calculators, can help to easily calculate the range of the amount of punishment corresponding to each degree of violation.

## Figures and Tables

**Table 1:** Wastewater grade and corresponding penalty range.

x Value Range	Wastewater Level	$\mathrm{Lower}\;\mathrm{Limit}\;\mathrm{of}\;\mathrm{Fine}\;y^{*}\;(\mathrm{Yuan})$	$\mathrm{Upper}\;\mathrm{Limit}\;\mathrm{of}\;\mathrm{Fine}\;\hat{y}$ (Yuan)	$f\left(x,y^{*}\right)$$=F\left(x,y^{*}\right)$ (Yuan)
x≤0.1	qualified	—	—	—
0.1<x≤1	level 1	37,142.857	104,000	33,428.57143
1<x≤2	level 2	56,842.105	108,000	25,578.94737
2<x≤3	level 3	70,000	112,000	21,000
3<x≤4	level 4	80,000	116,000	18,000
4<x≤5	level 5	88,235.294	120,000	15,882.35294
5<x≤6	Level 6	95,384.615	124,000	14,307.69231
6<x≤7	level 7	101,818.182	128,000	13,090.90909
7<x≤8	level 8	107,755.102	132,000	12,122.44898

$y^{*}\;and\;\hat{y}$ are the minimum and maximum penalty amount.

## Data Availability

The data supporting our research results are only the data in Table 1, which are all directly calculated according to Formulas (4) and (7), without reference to any literature.
